# Monitowapplication – an investigation of the usability during the first 18 months of commissioning in practice

**DOI:** 10.1192/j.eurpsy.2021.1622

**Published:** 2021-08-13

**Authors:** K. Tarp, T. Holmberg, A.M. Møller, M. Lichtenstein

**Affiliations:** 1 Research Unit For Telepsychiatry And E-mental Health, Centre For Telepsychiatry, Mental Health Services in the Region of Southern Denmark, Odense, Denmark; 2 Research Unit For Telepsychiatry And Emental Health, Centre for Telepsychiatry, Odense C, Denmark; 3 Health Promotion Research, Department Of Public Health, University of Southern Denmark, Odense, Denmark; 4 Research Unit For Telepsychiatry And E-mental Health, Mental Health Services in the Region of Southern Denmark, Odense, Denmark

**Keywords:** anxiety disorders, Anxiety monitoring, usability, App-based assessment

## Abstract

**Introduction:**

In cognitive behavioral treatment of anxiety disorders, registration of emotions and behavior is an important part of the intervention. Normally, paper and pencil is used but registrations on a mobile application such as MONARCA may be a useful alternative.

**Objectives:**

This study investigates the usability of MONARCA during the first 18 months of commissioning in practice.

**Methods:**

To explore the usability, semi-structured interviews were conducted with seven patients and three therapists, combined with data from a survey questionnaire where 10 patients and 12 therapists rated the usability of the app on the System Usability Scale. Participants were recruited from an outpatient clinic for affective disorders in The Mental Health Services in the Region of Southern Denmark.

**Results:**

Technical performance, time allocation, therapist effort, commitment, enthusiasm, and increased knowledge are imperative factors. Therapists and patients found that the benefits of registering emotions and behaviors on a mobile application were that it was easy for patients to remember to register daily, it was easy to gain an overview over symptom progress, and access to the registrations improved therapist’s ability to prepare sessions.

**Conclusions:**

Overall, the results from the interviews and survey indicated that both patients and therapists found MONARCA useful, but several improvement opportunities regarding application features and use in the treatment course were found.

**Disclosure:**

No significant relationships.

